# Antibiotics Trigger Host Innate Immune Response via Microbiota–Brain Communication in *C. elegans*

**DOI:** 10.3390/ijms25168866

**Published:** 2024-08-14

**Authors:** Yangyang Wu, Guanqun Li, Hongyun Tang

**Affiliations:** 1 College of Life Sciences, Zhejiang University, Hangzhou 310058, China; zsc@zju.edu.cn; 2 Key Laboratory of Growth Regulation and Translational Research of Zhejiang Province, School of Life Sciences, Westlake University, Hangzhou 310024, China; 3 Westlake Laboratory of Life Sciences and Biomedicine, Hangzhou 310024, China; 4 Institute of Biology, Westlake Institute for Advanced Study, Hangzhou 310024, China

**Keywords:** Q203 antibiotics, innate immune response, *cyp-14A4*, microbiota-brain communication, *C. elegans*

## Abstract

Besides their direct bactericidal effect, antibiotics have also been suggested to stimulate the host immune response to defend against pathogens. However, it remains unclear whether any antibiotics may stimulate the host immune response by affecting bacterial activity. In this study, reasoning that genetic mutations inhibit bacterial activities and, thereby, may mimic the effects of antibiotics, we performed genome-wide screening and identified 77 *E. coli* genes whose inactivation induces *C. elegans cyp-14A4*, representing an innate immune and detoxification response. Further analyses reveal that this host immune response can clearly be induced through either inactivating the *E. coli* respiratory chain via the bacterial *cyoB* mutation or using the antibiotic Q203, which is able to enhance host survival when encountering the pathogen *Pseudomonas aeruginosa*. Mechanistically, the innate immune response triggered by both the *cyoB* mutation and Q203 is found to depend on the host brain response, as evidenced by their reliance on the host neural gene *unc-13*, which is required for neurotransmitter release in head neurons. Therefore, our findings elucidate the critical involvement of the microbiota–brain axis in modulating the host immune response, providing mechanistic insights into the role of antibiotics in triggering the host immune response and, thus, facilitating host defense against pathogens.

## 1. Introduction

Pathogenic bacterial infections have severe impacts on human health [1,2,3]. At present, antibiotics are the primary method used to combat bacterial infections [4,5]. Antibiotics disrupt various essential processes in pathogens, including energy metabolism, cell wall synthesis, and protein synthesis [6]. In addition to their direct bactericidal effect, it has been suggested that antibiotics stimulate the host immune response to defend against pathogens [7,8,9]. For instance, nanoparticle-based antibiotics can promote bacterial lysis, leading to the release of immune-stimulating substances within the bacteria and consequently activating the host’s immune system [10]. However, the precise mechanisms through which antibiotics induce immune responses and the specific antibiotics involved in this process remain largely unclear. The innate immune response serves as the initial internal defense mechanism against pathogens, which is responsible for eliminating invading pathogens through processes such as inflammatory responses and detoxification [2,11]. Antibiotics that possess both direct bactericidal and immune-stimulatory effects may exhibit enhanced efficacy in defending against bacterial invasion while minimizing the development of antibiotic resistance [12,13,14]. Therefore, gaining a better understanding of how antibiotics can influence the innate immune response could offer valuable insights into the development of novel therapeutic approaches for bacterial infections.

The animal gut harbors a diverse population of commensal micro-organisms, such as *E. coli*, that play a crucial role in suppressing the growth of pathogens through competing for nutrients, producing antibacterial substances, and stimulating host immune responses [15,16,17]. Therefore, it is reasonable to hypothesize that certain antibiotics may alter the activity of gut commensal micro-organisms, thereby leading to the initiation of a host immune response. Identifying the specific bacterial processes that trigger the host immune response could provide valuable insights into identifying the antibiotics that affect the activity of gut bacteria such as to activate the host immune system. Moreover, exploring how gut microbes and antibiotics influence the host immune response may reveal previously unknown mechanisms for immune function modulation. For instance, given the common communication between gut microbes and the host brain, studying how gut commensal bacteria affect the host immune response may uncover the potential involvement of the nervous system in coordinating the immune response to these microbes, providing a new understanding of brain-mediated regulation of immunity [18,19].

The objective of our study was to identify bacterial processes that stimulate the innate immune response of the host, and utilize this knowledge to uncover potential antibiotics that modify the activity of gut commensal micro-organisms such as *E. coli* to initiate the host immune response. For this purpose, we conducted whole-genome screening and identified 77 *E. coli* genes that, when inactivated, can activate the detoxification and immune responses in the host organism *C. elegans*. Further analysis revealed that mutations in genes related to the bacterial respiratory chain and the use of the antibiotic Q203, which inhibits the respiratory chain, both significantly induced host immune responses and improved the host’s ability to survive against the pathogen *Pseudomonas aeruginosa* PA14. Additionally, we found that activation of the host immune response relies on the function of the nervous system, shedding light on the crucial role of neural regulation in the host’s immune response. In summary, our study facilitates a comprehensive understanding of the changes in bacterial activity that stimulate the host immune response and elucidates the important role of the nervous system in the host’s response to gut bacteria, thereby providing mechanistic insights into antibiotic-induced host immune responses.

## 2. Results

### 2.1. Genome-Wide Screening to Identify E. coli Genes Whose Inactivation Activates Detoxification and Immune Response in C. elegans

**Figure 1:**
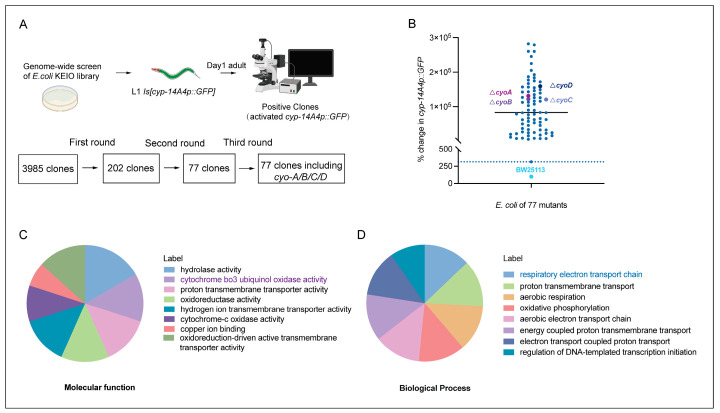
Genome-wide screening to identify *E. coli* genes whose deletion activates the detoxification and innate immune response in *C. elegans*. (**A**) Illustration of genome-wide screening for the identification of single-gene deletion mutants of *E. coli* that activate *cyp-14A4* expression in *C. elegans*, representing an innate immune and detoxification response. Through three rounds of screening, 77 single-gene deletions of *E. coli* mutants that enhance *cyp-14A4p::GFP* expression were identified. (**B**) Quantitative analysis was conducted to examine the expression of *cyp-14A4p::GFP* in response to the deletion of 77 individual genes of *E. coli*, as identified through the genome-wide screening. Each dot in the graph represents the total intensity of *cyp-14A4p::GFP* expression in *mgIs73[cyp-14A4p::GFP]* worms treated with indicated *E. coli* mutants carrying a single-gene deletion. To ensure accurate comparisons, the fluorescence intensity was normalized relative to the expression level observed in worms treated with wild-type *E. coli* BW25113 as the negative control, using a percentage scale n > 16 for each group. (**C**,**D**) The gene ontology analysis revealed that the 77 selected genes are enriched in various molecular functions (**C**) and biological processes (**D**) in *E. coli*, including cytochrome bo3 ubiquinol oxidase, which is involved in the respiratory chain.

Given that antibiotics exert bacteriostatic effects through the targeting of key processes in bacteria [6], bacterial gene mutations that inhibit bacterial activity potentially mimic the effects of antibiotics. Therefore, we performed genome-wide screening to identify *E. coli* genes whose inactivation induced an innate immune response in *C. elegans*, thereby providing clues for the identification of potential antibiotics that are able to trigger animal immune responses by affecting bacterial activity. The gene *cyp-14A4* encodes a cytochrome P450 enzyme, which is a heme protein that utilizes molecular oxygen to hydroxylate small molecule substrates as part of the detoxification process for toxins. Thus, a *cyp-14A4* promoter GFP fusion gene *[cyp-14A4p::GFP]* has been previously used to indicate both detoxification and innate immune response in *C. elegans* [20,21]. In our screening, we employed 3985 non-essential gene mutants from the *E. coli* Keio collection library [22], and examined their ability to induce the expression of *[cyp-14A4p::GFP]* in *C. elegans*. Following three rounds of screening, a total of 77 single-gene deletion mutants of *E. coli* were identified as inducing the expression of *cyp-14A4* in *C. elegans*, supporting the idea that inhibiting the bacterial biological processes involving these genes can trigger the host’s detoxification and immune responses (Figure 1A,B, Table S1).

To investigate the inhibition of bacterial processes that trigger host immune responses, a Gene Ontology (GO) analysis was performed on these 77 *E. coli* genes that activated the host *cyp-14A4*. The results of molecular function analyses showed that these genes encode various proteins, including hydrolases, oxidases, and transporters (Figure 1C). Furthermore, biological process analyses revealed that these genes were involved in various processes, including bacterial energy metabolism and transport, with an obvious enrichment in the electron transport chain process (Figure 1D). Therefore, our findings identified various bacterial processes that can affect the host immune response, providing a valuable resource for the identification of antibiotics that potentially stimulate the immune response of animals through the inhibiting of bacterial activity.

### 2.2. Q203 Antibiotic Potentially Activates the C. elegans Innate Immune Response and Detoxification Response through Targeting the Electron Transport Chain of Bacteria

**Figure 2:**
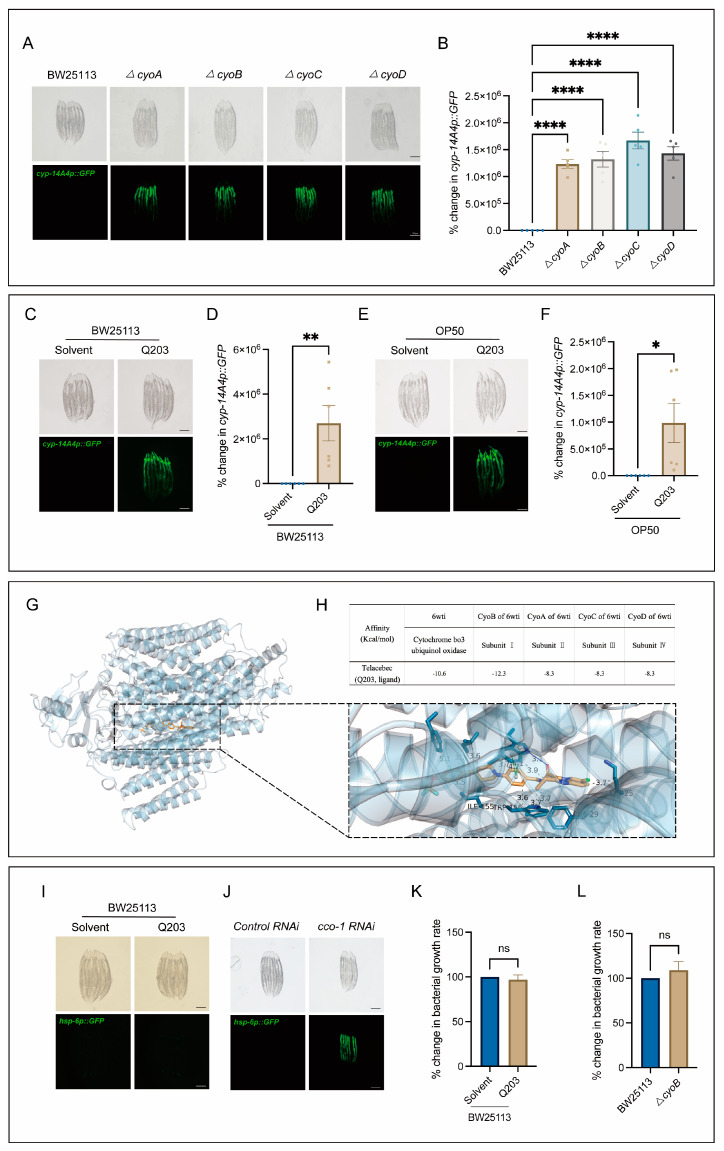
Q203 antibiotic activates detoxification and the innate immune response of *C. elegans*, possibly through inhibiting the electron transport chain of *E. coli*. (**A**,**B**) Images and bar graph indicate that *cyp-14A4p::GFP* expression is induced by *E. coli* mutants with a single-gene deletion in *cyoA/B/C/D* genes. Each dot represents the total intensity of *cyp-14A4p::GFP* expression in each group of worms. The percentage change in *cyp-14A4p::GFP* expression was determined by normalization with respect to the levels in worms treated with BW25113. The data are expressed as the mean ± SEM. Significance was assessed using a one-way ANOVA test (**** *p* < 0.0001), with a sample size of n > 30 for each group. The scale bar in the images corresponds to a length of 200 μm. (**C**,**D**) Microscopic images and bar graph showing the effects of the 500 μM Q203 + *E. coli* BW25113 on *cyp-14A4p::GFP* of *C. elegans*. The percentage change in *cyp-14A4p::GFP* expression was determined through normalization with respect to the levels in worms treated with BW25113 + Solvent. The data are expressed as the mean ± SEM. Significance was assessed using an unpaired *t*-test (** *p* < 0.01), with a sample size of n > 20 for each group. The scale bar in the images corresponds to a length of 200 μm. (**E**,**F**) Microscopic images and bar graph showing the effects of the 500 μM Q203 + *E. coli* OP50 on *cyp-14A4p::GFP* of *C. elegans*. The percentage change in *cyp-14A4p::GFP* expression was determined through normalization with respect to the levels in worms treated with OP50 + Solvent. The data are expressed as the mean ± SEM. Significance was assessed using an unpaired *t* test (* *p* < 0.05), with a sample size of n > 20 for each group. The scale bar in the images corresponds to a length of 200 μm. (**G**,**H**) Molecular docking analysis: The overall diagram shows that 6wti was selected as the receptor, while Q203 was chosen as the ligand. In the local diagram, hydrogen bonding, hydrophobic interactions, and π-π stacking interactions are represented by blue solid lines, gray dashed lines, and green dashed lines, respectively (**G**). The table presents the binding energies for Q203 binding with 6wti, CyoB, CyoA, CyoC, and CyoD in the molecular docking analysis (**H**). (**I**,**J**) Exogenous addition of Q203 had no induction effect on *zcIs13[hsp-6p::GFP]*, while *cco-1* RNAi served as the positive control, effectively inducing the activation of *hsp-6*. (**K**) Exogenous addition of Q203 had no significant effect on bacterial growth. The percentage change in bacterial growth rate was calculated through normalization with respect to the levels in BW25113 treated with Solvent. The data are expressed as the mean ± SEM. Significance was assessed using unpaired *t*-test (*p* > 0.05, ns), with a sample size of n = 3 for each group. (**L**) No significant difference in growth rate was observed between *ΔcyoB E. coli* and wild-type *E. coli* BW25113. The percentage change in bacterial growth rate was calculated through normalization with respect to the levels in BW25113. The data are expressed as the mean ± SEM. Significance was assessed using an unpaired *t*-test (*p* > 0.05, ns), with a sample size of n = 3 for each group.

Next, we conducted tests to determine the potential usefulness of the bacterial mutations obtained through our screening process, in terms of identifying antibiotics that could elicit a host immune response. Notably, there was a clear enrichment of mutations in genes associated with the bacterial respiratory chain (Figure 1C), which are often targeted for antibiotic development [6,23]. Thus, we first performed independent experiments to verify the effect of inactivation of the bacterial respiratory chain on triggering host immune response, and then tested whether antibiotics targeting this bacterial process may also trigger the host immune response. As expected, mutations of *cyo-A/-B/-C/-D* genes, encoding the subunits of cytochrome bo3 ubiquinol oxidase [24,25], had a significant inducing effect on the expression of *cyp-14A4* (Figure 2A,B). Furthermore, Q203, as a novel antibiotic targeting the respiratory chain of *Mycobacterium tuberculosis* to inhibit its growth [26,27], was used to test its ability in stimulating the *C. elegans* immune response through mimicking the effect of mutations of *cyo-A/-B/-C/-D* genes. Indeed, the induction of *mgIs73[cyp-14A4p::GFP]* was evident in worms cultured on wild-type BW25113 *E. coli* supplemented with Q203 in a concentration-dependent manner, in contrast to those treated with DMSO as a control (Figure 2C,D, Figure S1). Moreover, *mgIs73[cyp-14A4p::GFP]* was also significantly triggered in the worms treated with OP50 *E. coli* mixed with Q203 but not with DMSO (Figure 2E,F), supporting the conclusion that the immune response-activating effect of Q203 is not influenced by the *E. coli* strain. Additionally, our quantitative PCR (qPCR) analyses revealed that either *ΔcyoB E. coli* or Q203 treatment can upregulate the infection response gene *irg-1* and the antimicrobial peptide-encoding gene *nlp-29* (Figure S2), indicating their ability to trigger a general immune response. These results reveal that the screening of bacterial gene mutations for host immune activation is useful for identifying potential antibiotics that can activate the animal’s immune response.

Additionally, through employing macromolecular docking analyses [28], we found that Q203 exhibits a strong binding affinity for *E. coli* cytochrome bo3 ubiquinol oxidase, with a binding energy of −10.6 kcal/mol (Figure 2G,H). Specifically, a comprehensive analysis of the binding energies between the four subunits containing cytochrome bo3 ubiquinol oxidase and Q203 was conducted. The results demonstrated that subunit I, encoded by *cyoB*, displays the strongest affinity towards Q203 with a binding energy of −12.3 kcal/mol (Figure 2H), supporting a high specificity for Q203 to target CyoB. Moreover, we found that Q203 supplementation did not induce the expression of the *C. elegans zcIs13[hsp-6p::GFP]* reporter (Figure 2I)—a widely used indicator of mitochondrial functional state [29]—in contrast to the obvious induction by *cco-1(RNAi)*, which served as the positive control (Figure 2J). This result thus suggests that this antibiotic may not act on the worm mitochondria to trigger an immune response. In addition, our results rule out the possibility that Q203 triggers the *C. elegans* immune response due to its bacteria-killing effect. We found that Q203 showed no effect on the proliferation of *E. coli*, similar to the effect of *ΔcyoB* mutation (Figure 2K,L), which suggests that other pathways redundant with *cyoB* may exist to maintain bacterial respiration, and that Q203 and *cyoB* mutation may affect certain non-essential bacterial activities to trigger the host immune response. Taken together, our results suggest that the host immune response could be effectively induced through inactivating the *E. coli* respiratory chain via both the bacterial *cyoB* mutation and antibiotic Q203 treatment.

### 2.3. Host Brain Activity Is Required for ΔcyoB E. coli and Q203 to Activate C. elegans Detoxification and Immune Response

**Figure 3:**
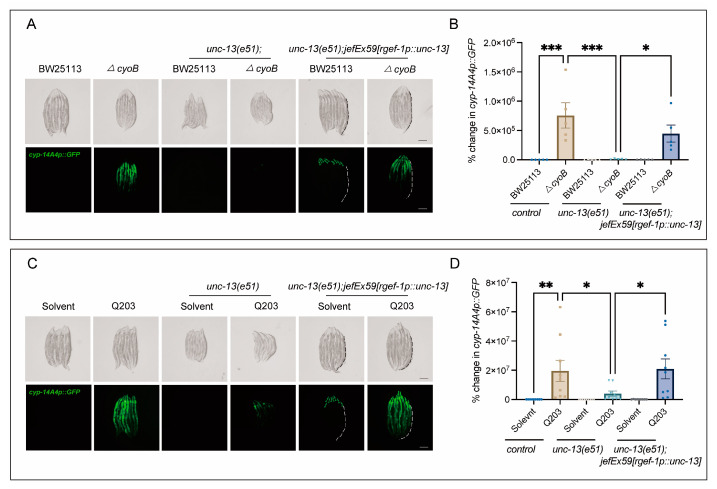
Neural function is required for the activation of *cyp-14A4* expression by *ΔcyoB E. coli* and Q203. (**A**,**B**) Microscopic images and bar graphs showing that UNC-13 is required for the activation of *cyp-14A4* expression induced by *ΔcyoB E. coli*. The *unc-13(e51)* loss-of-function mutant was used to analyze the role of UNC-13 in mediating the bacteria-triggered *cyp-14A4* response. The *unc-13(e51);jefEx59[rgef-1p::unc-13];mgIs73[cyp-14A4p::GFP]* strain of worms was utilized, wherein *cyp-14A4p::GFP* is expressed, and *unc-13* is specifically expressed in neurons. The change in *cyp-14A4p::GFP* fluorescence in the intestine was quantified through calculating the percentage change, normalized to the fluorescence levels observed in *cyp-14A4p::GFP* control animals treated with BW25113. The dashed line represents the GFP signal in the intestine. The data are expressed as the mean ± SEM. Significance was assessed using a two-way ANOVA test (* *p* < 0.05, *** *p* < 0.001), with a sample size of n > 20 for each group. The scale bar in the images corresponds to a length of 200 μm. (**C**,**D**) Microscopic images and bar graphs showing that UNC-13 is required for the activation of *cyp-14A4* expression induced by 500 μM Q203. The change in *cyp-14A4p::GFP* fluorescence of the intestine was quantified through calculating the percentage change, normalized to the fluorescence levels observed in *cyp-14A4p::GFP* control animals treated with BW25113 + Solvent. The dashed line represents the GFP signal in the intestine. The data are expressed as the mean ± SEM. Significance was assessed using a two-way ANOVA test (* *p* < 0.05, ** *p* < 0.01), with a sample size of n > 20 for each group. The scale bar in the images corresponds to a length of 200 μm.

It has been suggested that the brain plays a crucial role in monitoring gut bacterial changes [19,30,31,32]. Therefore, we tested whether the host’s brain may be required for the activation of bacteria-induced innate immune responses. Strikingly, we found that disrupting the release of neurotransmitters through the loss of function of *unc-13*—encoding diacylglycerol-binding proteins that are required for presynaptic fusion of synaptic vesicles with the plasma membrane [33]—significantly inhibited the activation of *cyp-14A4* induced by *ΔcyoB E. coli*, compared to the obvious induction of *cyp-14A4* observed in the wild-type worms treated with the *ΔcyoB E. coli* (Figure 3A,B). Importantly, the activation of *cyp-14A4* induced by the *ΔcyoB E. coli* was restored in the *unc-13(e51)* through expressing *unc-13* with the *rgef-1* pan-neuron promoter [34], thereby further elucidating the neural function of UNC-13 in mediating the host immune response to bacterial change (Figure 3A,B). Similarly, we found that *unc-13(e51)* also repressed the Q203-induced *cyp-14A4*, where this repression could be reversed via neural expression of UNC-13 (Figure 3C,D). These findings revealed that UNC-13-mediated neurotransmitter release is required for the activation of host *cyp-14A4* via Q203 and *ΔcyoB E. coli*, in alignment with the implication that Q203 induces a detoxification and immune response through repressing the *E. coli* electron transport chain.

### 2.4. Brain-Mediated Activation of Immune Response by ΔcyoB E. coli and Q203 Enhances the Ability of C. elegans to Resist Pathogenic Invasion

**Figure 4:**
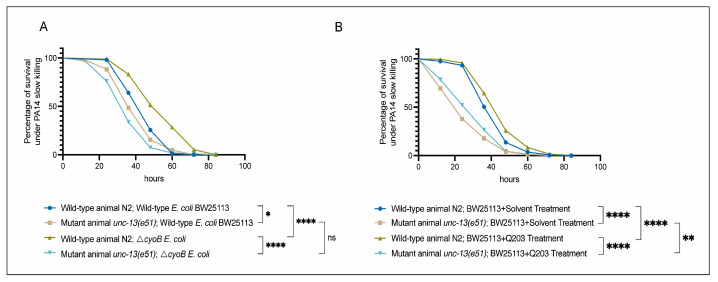
Brain-mediated activation of the innate immune response by *ΔcyoB E. coli* and Q203 enhances resistance to PA14. (**A**) Survival curve results showing that *unc-13* mediates the activation of *ΔcyoB E. coli*-induced innate immune responses, improving the ability of animals to resist pathogenic bacterial PA14 virulence. The survival rates of the *unc-13(e51)* and wild-type N2 animals, either with *ΔcyoB E. coli* or BW25113 *E. coli* pre-treatment, were compared after exposure to PA14. Significance was assessed using Log-rank test (* *p* < 0.05, **** *p* < 0.0001, ns > 0.05), with a sample size of n > 90 for each group. (**B**) Survival curve results showing that *unc-13* mediates the activation of Q203-induced innate immune responses, improving the ability of animals to resist pathogenic bacterial PA14 virulence. The survival rates of the *unc-13(e51)* and wild-type N2 animals, either with BW25113 + 500 μM Q203 or BW25113 + Solvent pre-treatment, were compared after exposure to PA14. Significance was assessed using a Log-rank test (** *p* < 0.01, **** *p* < 0.0001), with a sample size of n > 200 for each group.

**Figure 5:**
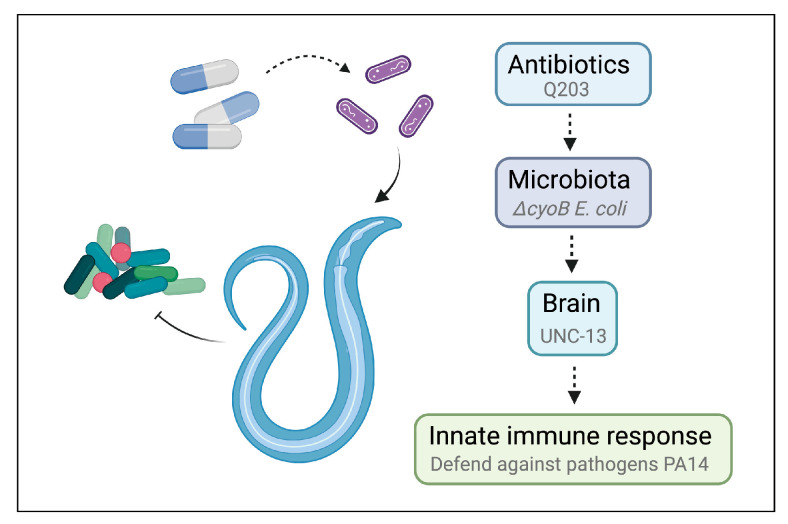
Schematic model for antibiotics triggering host innate immune response via microbiota–brain communication in *C. elegans*.

Next, we tested the role of UNC-13-mediated activation of the immune response in defending against a common pathogenic bacterium, *Pseudomonas aeruginosa* PA14 [35,36,37]. First, we evaluated the sensitivity of *unc-13(e51)* worms to pathogenic infection with PA14 through measuring the survival rate of these mutant animals in a PA14 slow-killing assay [35]. We found that, when grown on wild-type BW25113 *E. coli*, the *unc-13(e51)* mutant worms exhibited reduced survival rates in the PA14 slow-killing assay compared to the wild-type N2 (Figure 4A). This result indicates the important role of the nervous system in the survival response of *C. elegans* to PA14 infection. Furthermore, we found that the activation of the immune response via *ΔcyoB E. coli* and Q203 was able to improve the survival of the N2 worms exposed to PA14, as evidenced by the observation that N2 adult worms pre-treated with *ΔcyoB E. coli* and Q203 exhibited increased survival rates in the PA14 slow-killing assay compared to those pre-treated with wild-type *E. coli* BW25113 or with BW25113 + Solvent groups (Figure 4A,B). Importantly, the enhanced survival rate of worms pre-treated with *ΔcyoB E. coli* and Q203 in the PA14 slow-killing assay was repressed by the *unc-13(e51)* mutation (Figure 4A,B). Therefore, the brain-mediated activation of the immune response is important for the Q203 antibiotic to exert an effect on the resistance to pathogen invasion in *C. elegans*. Additionally, there is no significant difference between the survival rate of *unc-13(e51)* mutant worms on PA14 following pre-treatment with BW25113 or *ΔcyoB E. coli*, while the survival rate of *unc-13(e51)* worms pre-treated with Q203 was significantly higher than those pre-treated with Solvent (Figure 4A,B). This suggests that, unlike the immune response induced by *ΔcyoB E. coli*, the response induced by Q203 is not entirely dependent on *unc-13* regulation. There is potential for partial enhancement of the immune response through alternative pathways, which is consistent with the finding that *unc-13(e51)* partially inhibits the activation of *cyp-14A4* induced by Q203. Overall, the brain-mediated activation of the immune response by *ΔcyoB E. coli* and Q203 enhances *C. elegans’* ability to resist pathogenic invasion (Figure 5).

## 3. Discussion

Whether antibiotics can trigger the host response through affecting gut bacterial activity is an important question. In this study, we identified 77 *E. coli* genes whose inactivation can induce a host immune response, providing clues for the discovery of potential antibiotics that can trigger the host immune response through affecting bacterial activity. Further analysis revealed that the bacterial *cyoB* mutation and the antibiotic Q203, which both inhibit the bacterial respiratory chain, could effectively induce innate immunity and detoxification reactions in *C. elegans*. Importantly, our study revealed that this activation of the immune response depends on the function of the nervous system. Therefore, this study elucidates the critical role of the microbe–brain axis in modulating the host immune response, providing insights into the mechanisms by which antibiotics trigger host immune responses to enhance the host’s defense against pathogens.

Previous research has primarily focused on the bactericidal effects of antibiotics, such as interference with DNA synthesis and inhibition of cell wall synthesis [6]. However, recent studies have highlighted the potential of antibiotics to modulate the host’s immune response. Some antibiotics have been found to hinder immune responses, thus increasing susceptibility to infection [38]; for example, vancomycin, neomycin, and metronidazole have been shown to effectively reduce the expression of REG3γ, a C-type lectin [39]. Additionally, treatment with penicillin and streptomycin has been observed to decrease the population of Th17 cells within the mouse intestine [40]. Nevertheless, the impacts of different antibiotics on host immunity do not always yield negative outcomes. To the contrary, certain studies have suggested that antibiotics can activate the host immune response, thereby synergistically inhibiting pathogen invasion [41]. Our study revealed that the antibiotic Q203 induces the host’s innate immune response and enhances its ability to resist pathogen infection, thus supporting the synergistic role of antibiotics and the host immune system in combating pathogens.

A wealth of evidence indicates that the gut microbiota can influence the immune response of the host, which is crucial in determining susceptibility to intestinal pathogen infections and the development of intestinal diseases [4,42]. Antibiotic treatment during the progression of these infections indirectly affects the host’s immune system through altering the activity and metabolic byproducts of the intestinal microbiota, thus impacting the immune response [4,43,44]. Nevertheless, the exact molecular mechanisms responsible for these effects remain unclear. One prominent approach for the development of antibiotics involves inhibiting the proliferation and invasion of microbiota through targeting their respiratory chain [23]. Q203, a compound designed to combat *Mycobacterium tuberculosis* infections, operates on this principle by blocking binding of the cytochrome bcc complex to its substrate, thereby impeding the aerobic respiratory pathway of this obligate aerobe [23,27]. Notably, our findings indicated that neither Q203 nor *ΔcyoB* inhibited the amplification of *E. coli*, possibly because *E. coli* is a facultative anaerobic bacterium and there exist other redundant pathways to compensate for the loss of *cyoB* [45]. Overall, our study supports the notion that Q203 induces the indirect activation of innate immune responses in *C. elegans* through the modification of respiratory chain activity in *E. coli*.

Regarding the molecular mechanisms, we demonstrated that the nervous system plays a crucial role in mediating the activation of host immune defense by antibiotics. According to our findings, *unc-13(e51)* mutant worms, which have defects in neurotransmitter synaptic transmission [33], inhibited the expression of *cyp-14A4* induced by antibiotics. This provides evidence that blocking neurotransmitter pathways can suppress the innate immune response in worms. Furthermore, we observed that the ability of *unc-13(e51)* mutant animals to resist pathogenic bacteria PA14 was reduced when compared to the wild-type worm N2, which is consistent with previous research [37]. Therefore, the above findings suggest that the nervous system of the host mediates the activation of antibiotic-induced innate immune responses, enhancing resistance against pathogenic invasion.

Our research uncovered the substantial influences of bacterial gene inactivation and antibiotics on the stimulation of the innate immune response. Nevertheless, several limitations within this study necessitate further investigation in future research. One area for exploration is testing whether the immune response induced by Q203 or *ΔcyoB E. coli* correlates with nutritional status, such as iron deficiency. The overlap in gene lists identified through whole-genome screening and the study by Zhang suggest that iron deficiency caused by mutant bacteria may trigger the activation of the host’s immune response [46]. Recent epidemiological association analyses show that intracellular free iron levels are inversely correlated with infection resistance, such as iron supplementation exacerbating pathogenic bacterial infections [47]. Consequently, whether the decline in labile iron levels mediates the augmented efficacy of antibiotics or bacterial mutants against pathogenic infection, as observed in our study, warrants further investigation. Another area for exploration is the role of UNC-13 in synaptic signal transmission for multiple neurotransmitters. Specifically, which neurotransmitters are involved in this process remains to be studied. Additionally, while this study primarily focused on the impact on innate immunity, it failed to comprehensively examine the associated effects on adaptive immunity. The recognition of pathogens and the establishment of immune memory are crucial aspects of long-term immune defense in the human body. Therefore, future research should consider utilizing mammalian models with adaptive immune systems to validate the reported findings. Through conducting extensive research, we can acquire a deeper understanding of the role of antibiotics in immune regulation, ultimately helping to tackle the pressing issue of antibiotic resistance and facilitate the development of novel anti-infective therapies.

## 4. Materials and Methods

### 4.1. Strains and Maintenance

The *C. elegans* strains MAT207 *[unc-13(e51)];mgIs73[cyp-14A4p::GFP]* and MAT223 *[unc-13(e51)];mgIs73[cyp-14A4p::GFP];jefEx59[rgef-1p::UNC-13]* were created in our laboratory. The other strains GR2250 *mgIs73[cyp-14A4p::GFP]*, MT7929 *[unc-13(e51)]*, SJ4100 *zcIs13 [hsp-6p::GFP]* and N2 used in this study were obtained from Caenorhabditis Genetics Center (CGC, Minneapolis, MN, USA). Additionally, the bacterial strains used in this study were *E. coli* OP50, obtained from CGC, and *E. coli* K-12 from Keio Knockout library collection (OEC4988) and the corresponding wild-type parent strain BW25113 (OEC5042), as well as the ORF RNAi Collection (RCE1181), all of them obtained from Dharmacon (Lafayette, CO, USA). All *C. elegans* strains were cultured and maintained under standard conditions at 20 °C.

### 4.2. Screening for E. coli K-12 Mutants That Activate cyp-14A4 Expression of C. elegans and GO Analysis

*cyp-14A4*, as a member of the cytochrome P450 family, is classified as one of the genes related to detoxification and immunity. We investigated the activation of *cyp-14A4* expression by screening for individual gene deletions using the Keio library collection (OEC4988), which consists of deletion strains for each of the non-essential 3985 genes, with the aim of assessing the effect of bacterial variations on innate immune reactions in worms through GO enrichment analysis.

Specifically, three rounds of screening were performed to identify bacterial gene deletions that enhance *cyp-14A4p::GFP* expression. In each round, the wild-type BW25113 *E. coli* and each *E. coli* mutant strain were cultured overnight in liquid LB medium (200 μL), with or without 100 μg/mL kanamycin, in a 96-well plate. The cultures were subsequently inoculated onto nematode-growth media (NGM) plates with or without 50 μg/mL kanamycin. Synchronized L1-stage worms were then cultured and, when they reached the day-1 adult stage, the fluorescence of *cyp-14A4p::GFP* was assessed using a Nikon SMZ18 microscope (Nikon Corporation, TKY, Japan). Throughout the screening process, *cyp-14A4p::GFP* worms fed with wild-type *E. coli* BW25113 served as parallel negative controls. In the second round of screening, the 202 bacterial clones identified in the first round as increasing *cyp-14A4p::GFP* expression were re-examined, resulting in the selection of 77 positive clones for further analysis. In the subsequent third round of screening, these 77 clones were again included to confirm their status as positive clones. Across all three independent experiments, it was consistently observed that these 77 positive clones significantly enhanced the expression of *cyp-14A4p::GFP*. Finally, in order to explore which bacterial processes influence the host’s *cyp-14A4p::GFP* expression, GO enrichment analysis was performed on these 77 mutant genes, and corresponding images were generated using the Prism software (v10.0).

### 4.3. Q203 Supplementation

Q203 (MedChemExpress Cat# HY-101040, Monmouth Junction, NJ, USA) was solubilized in DMSO and added to OP50 *E. coli* or BW25113 *E. coli*, resulting in a final concentration of 1 μM, 20 μM, 100 μM, 500 μM, and 1 mM. As a negative control, an equivalent volume of the DMSO solvent was added to OP50 *E. coli* or BW25113 *E. coli*, and then seeded at NGM plates. The mixture of bacteria and Q203 was then dried in a laminar flow hood and subsequently incubated at 37 °C for 16 h. To assess the impact of Q203 on the indicated phenotypes, L1-stage worms were cultured with the indicated Q203 supplements and bacterial treatments. Until reaching the day-1 adult stage, the indicated phenotypes were scored as described.

### 4.4. Imaging and Analysis of the Expression of Transgenic Reporter cyp-14A4p::GFP

For microscopic imaging of *mgIs73 [cyp-14A4p::GFP]*, approximately 100 L1-stage reporter worms were cultured with the indicated treatment until reaching the day-1 adult stage. To examine the expression of *cyp-14A4p::GFP* resulting from the indicated treatments, individual worms were selected and anesthetized on an NGM plate. Images of 8 worms per group were then captured using a Nikon SMZ18 microscope. The acquired images were subsequently analyzed using the ImageJ software 1.54g to quantify the fluorescence intensity.

### 4.5. qPCR Analyses

Around 400 young adult worms, with specified genotypes and treatments, were collected for RNA extraction using the MicroElute Total RNA Kit (Omega R6831, Omega Engineering, Norwalk, CT, USA). Purified RNA samples were treated with the One-Step gDNA Remover (Transgen AT311, Beijing, China) to eliminate potential genomic DNA contamination. Thereafter, the RNA was reverse transcribed into cDNA using the cDNA Synthesis SuperMix Kit (Transgen AT311, Beijing, China). qPCR was then conducted with primers specific to the indicated genes and Universal qPCR Master Mix (BioLabs, Ipswich, MA, USA). The mRNA levels of the designated genes were normalized against rpl-26 transcripts. The qPCR primers employed were shown in Table 1.

### 4.6. RNA Interference in C. elegans

HT115 *E coli*, expressing *cco-1* dsRNA, were initially cultured in LB medium supplemented with 50 μg/mL ampicillin (Amp) overnight. These bacteria were then inoculated onto NGM plates containing 2 mM isopropyl β-D-1-thiogalactopyranoside (IPTG) to further induce *cco-1* dsRNA production. Synchronized *zcIs13[hsp-6p::GFP]* L1-stage worms were seeded onto these RNAi plates to knock down *cco-1* expression. The induction of *hsp-6p::GFP* in the worms was assessed using a fluorescence microscope when the worms reached the day-1 adult stage.

### 4.7. Quantification of Bacterial Growth

The Q203 mixture was added to LB, adjusting the final concentration to 500 μM. For the control group, LB broth supplemented with an equal volume of DMSO was utilized. Subsequently, an equal amount of BW25113 bacteria was introduced into both the LB-Q203 and LB-DMSO mixtures as the seed culture. The cultures were incubated at 37 °C for a duration of 8 h, monitoring their progression into the exponential growth phase. Upon reaching this phase, the samples were retrieved, adequately diluted, and inoculated onto agar plates. An additional 12-h incubation period at 37 °C was followed by quantification of the number of resulting colonies. To compare the bacterial growth rates of *ΔcyoB E. coli* and BW25113, an equivalent and limited quantity of bacteria was introduced into LB broth to establish a seed culture. The remaining steps were consistent with the aforementioned ones.

### 4.8. Generation of Transgenic Worm Strains

The transgenes containing the *[rgef-1p::UNC-13::unc-54 3′UTR]* construct were utilized to specifically express *unc-13* in the *unc-13(e51)* background, thereby rescuing the neurotransmitter release function in pan-neurons. In detail, the promoter region of *rgef-1* was employed to drive the expression of these genes within pan-neurons. The 5′GAATTCCTGCAGCCCTTTCCGTCAATTCTACCTCCCCAAT3′ and 5′ATCCATGGATCCCCCCGTCGTCGTCGTCGATGC3′ primers were employed to amplify the *rgef-1* promoter through PCR, while the 5′ATGGATGACGTTGGAGATTACAA3′ and 5′CTATGTTCGATTGATGTTTTGACTTGA3′ primers were used to amplify *unc-13* cDNA. A DNA mixture was prepared for microinjection, comprising a co-injection marker *myo-2p::GFP* at a concentration of 20 ng/μL and a plasmid containing the specified genes at a concentration of 50 ng/μL. Then, worms carrying the *rgef-1p::UNC-13::unc-54 3′UTR* extrachromosomal array were obtained from microinjections.

### 4.9. PA14 Slow-Killing Assay

The PA14 slow-killing assay was chosen to evaluate the resistance of worms to pathogen-induced mortality, as previously described [35]. Briefly, overnight-cultured *Pseudomonas aeruginosa* PA14 was inoculated onto 35 mm diameter plates containing Slow Killing (SK) agar media. The SK agar media was prepared by mixing 3.5 g of Bacto-Peptone, 3 g of NaCl, and 17 g of Bacto-Agar in 1 L of distilled water, followed by high-pressure sterilization. After sterilization, 1 mL of 1M MgSO_4_, 25 mL of 1M KH_2_PO_4_, 1 mL of 1M CaCl_2_, and 1 mL of 5 mg/mL cholesterol in ethanol were added. The plates were fully coated with the inoculum of PA14 and air-dried at room temperature, then incubated at 37 °C for 24 h. Subsequently, the plates were transferred to a temperature of 25 °C and incubated for an additional 24 h. After at least 1 h of cooling at room temperature, the plates were used for the PA14 slow-killing assay. Synchronized young adult-stage worms were transferred onto the PA14-infused plates and cultured at 20 °C. The survival of the worms was monitored at 12-h intervals. Worms on the bacterial lawn that displayed no response or movement when gently touched with a platinum wire on either their nose or tail were categorized as dead and counted. All survival rate determinations were repeated at least three times, with a minimum of 20 worms counted in each experiment.

### 4.10. Macromolecular Docking

Molecular docking is a technique utilized to predict the optimal binding arrangement between different molecules. In this study, we employed a semi-flexible docking approach to form stable complexes and performed molecular docking of a ligand (Q203, PubChem CID: 68234908) with a receptor (cytochrome bo3 ubiquinol oxidase, RCSB ID: 6wti) using the AutoDock Vina 1.1.2 software [48]. In detail, 6wti was pre-processed using PyMol 2.3.3, including opening the sequence, removing water molecules and unnecessary ligands, and adding hydrogen atoms. Subsequently, AutoDock Tools 1.5.6 was used to generate PDBQT files for both Q203 and 6wti, in order to prepare for docking simulations. The size of the docking box was set to the values in the provided table, with a grid spacing of 1.00 Å. Other parameters were kept at their default values except for the coordinates, which were set as described below. The conformation with the lowest binding energy and highest clustering frequency was chosen as the most potential binding mode between Q203 and 6wti, out of the nine optimal docking conformations. This conformation was then subjected to analysis of interaction forces and bond lengths using Plip. The docking results were further visualized using PyMol 2.3.3, enabling a comprehensive examination of ligand–receptor binding and subsequent analysis of the stability and intermolecular interactions of the complex. Additionally, the docking processes of CyoA, CyoB, CyoC, and CyoD with Q203 were consistent with those of 6wti. The docking boxes and coordinates (x, y, z) were shown in Table 2.

### 4.11. Statistical Analysis

We conducted at least three independent experiments to ensure the robustness of our findings. In the Figures and Results sections, the sample size, represented by “n”, refers to the number of worms analyzed in each group for each experiment. Statistical analyses were performed using GraphPad Prism software 10.2.0. For comparisons between two groups, we utilized Student’s *t*-test, while one- or two-way ANOVA tests were employed for comparisons among multiple groups. Data are presented as the standard error of the mean (SEM). Significance levels are denoted as follows: *, *p* < 0.05; **, *p* < 0.01; ***, *p* < 0.001; ****, *p* < 0.0001.

## Figures and Tables

**Table 1:** qPCR primer sequences.

Gene Name	Primers	Sequence
*rpl-26*	primer F	CTTCGAAGGTCGCTATCACCA
	primer R	TTGACGGTCTCGTCAGTGTG
*cyp-14A4*	primer F	CGTTTGTAACGCAAGGCGAA
	primer R	CGTTTCCAACGCAAAGCTGA
*nlp-29*	primer F	TATGGAAGAGGATATGGAGGATATG
	primer R	TCCATGTATTTACTTTCCCCATCC
*irg-1*	primer F	TGAAACTTGTGGAGGCCTCA
	primer R	TGGCATCTAGTTTCCAGGCT

**Table 2:** The parameters about the size and coordinates of the docking box.

	Docking Box	x	y	z
6wti	101 Å × 101 Å × 101 Å	161.597	168.208	157.094
CyoB	62 Å × 70 Å × 60 Å	170.031	161.868	151.459
CyoA	106 Å × 64 Å × 56 Å	160.696	182.206	141.716
CyoC	88 Å × 64 Å × 56 Å	160.696	170.206	180.716
CyoD	81 Å × 56 Å × 65 Å	160.696	180.206	180.716

## Data Availability

The original contributions presented in the study are included in the article/Supplementary Materials, further inquiries can be directed to the corresponding authors.

## Supplementary Materials

The following supporting information can be downloaded at: https://www.mdpi.com/article/10.3390/ijms25168866/s1.

## References

[1] GBD 2019 Antimicrobial Resistance Collaborators (2022). Global mortality associated with 33 bacterial pathogens in 2019: A systematic analysis for the Global Burden of Disease Study 2019. Lancet.

[2] Jo E.K. (2019). Interplay between host and pathogen: Immune defense and beyond. Exp. Mol. Med..

[3] Cao P., Fleming D., Moustafa D.A., Dolan S.K., Szymanik K.H., Redman W.K., Ramos A., Diggle F.L., Sullivan C.S., Goldberg J.B. (2023). A *Pseudomonas aeruginosa* small RNA regulates chronic and acute infection. Nature.

[4] Ubeda C., Pamer E.G. (2012). Antibiotics, microbiota, and immune defense. Trends Immunol..

[5] Lewis K. (2020). The science of antibiotic discovery. Cell.

[6] Kohanski M.A., Dwyer D.J., Collins J.J. (2010). How antibiotics kill bacteria: From targets to networks. Nat. Rev. Microbiol..

[7] Berti A., Rose W., Nizet V., Sakoulas G. (2020). Antibiotics and innate immunity: A cooperative effort toward the successful treatment of infections. Open Forum Infect. Dis..

[8] Hodille E., Rose W., Diep B.A., Goutelle S., Lina G., Dumitrescu O. (2017). The role of antibiotics in modulating virulence in *Staphylococcus aureus*. Clin. Microbiol. Rev..

[9] Craven R.R., Gao X., Allen I.C., Gris D., Bubeck Wardenburg J., McElvania-Tekippe E., Ting J.P., Duncan J.A. (2009). *Staphylococcus aureus* alpha-hemolysin activates the NLRP3-inflammasome in human and mouse monocytic cells. PLoS ONE.

[10] Li R.S., Liu J., Wen C., Shi Y., Ling J., Cao Q., Wang L., Shi H., Huang C.Z., Li N. (2023). Transformable nano-antibiotics for mechanotherapy and immune activation against drug-resistant Gram-negative bacteria. Sci. Adv..

[11] Zhou Z., Xu M.J., Gao B. (2016). Hepatocytes: A key cell type for innate immunity. Cell. Mol. Immunol..

[12] Olson J., Nonejuie P., Dam Q., Dhand A., Pogliano J., Yeaman M.R., Hensler M.E., Bayer A.S., Nizet V. (2014). Nafcillin enhances innate immune-mediated killing of methicillin-resistant. J. Mol. Med..

[13] Dhand A., Bayer A.S., Pogliano J., Yang S.J., Bolaris M., Nizet V., Wang G., Sakoulas G. (2011). Use of antistaphylococcal β-Lactams to increase daptomycin activity in eradicating persistent bacteremia due to methicillin-resistant: Role of enhanced daptomycin binding. Clin. Infect. Dis..

[14] Dhand A., Sakoulas G. (2014). Daptomycin in combination with other antibiotics for the treatment of complicated methicillin-resistant bacteremia. Clin. Ther..

[15] Yang J.H., Bhargava P., McCloskey D., Mao N., Palsson B.O., Collins J.J. (2017). Antibiotic-induced changes to the host metabolic environment inhibit drug efficacy and alter immune function. Cell Host Microbe.

[16] Kelly D., Campbell J.I., King T.P., Grant G., Jansson E.A., Coutts A.G., Pettersson S., Conway S. (2004). Commensal anaerobic gut bacteria attenuate inflammation by regulating nuclear-cytoplasmic shuttling of PPAR-γ and RelA. Nat. Immunol..

[17] Pickard J.M., Zeng M.Y., Caruso R., Núñez G. (2017). Gut microbiota: Role in pathogen colonization, immune responses, and inflammatory disease. Immunol. Rev..

[18] Poojara L., Acharya D.K., Patel J., Rawal R. (2022). Gut–Brain Axis: Role of the Gut Microbiome on Human Health in Microbiome-Gut-Brain Axis.

[19] Morais L.H., Schreiber H.L.t., Mazmanian S.K. (2021). The gut microbiota-brain axis in behaviour and brain disorders. Nat. Rev. Microbiol..

[20] Mao K., Ji F., Breen P., Sewell A., Han M., Sadreyev R., Ruvkun G. (2019). Mitochondrial dysfunction in activates mitochondrial relocalization and nuclear hormone receptor-dependent detoxification genes. Cell Metab..

[21] Lim S.Y.M., Pan Y., Alshagga M., Lim W., Cin K., Alshehade S.A., Alshawsh M. (2024). CYP14 family in *Caenorhabditis elegans*: Mitochondrial function, detoxification, and lifespan. J. Appl. Toxicol..

[22] Baba T., Ara T., Hasegawa M., Takai Y., Okumura Y., Baba M., Datsenko K.A., Tomita M., Wanner B.L., Mori H. (2006). Construction of *Escherichia coli* K-12 in-frame, single-gene knockout mutants: The Keio collection. Mol. Syst. Biol..

[23] Zhou S., Wang W., Zhou X., Zhang Y., Lai Y., Tang Y., Xu J., Li D., Lin J., Yang X. (2021). Structure of cytochrome in complex with Q203 and TB47, two anti-TB drug candidates. elife.

[24] Chepuri V., Lemieux L., Au D.C., Gennis R.B. (1990). The sequence of the Cyo operon indicates substantial structural similarities between the cytochrome-o ubiquinol oxidase of *Escherichia coli* and the aa3-type family of cytochrome-c oxidases. J. Biol. Chem..

[25] Nakamura H., Yamato I., Anraku Y., Lemieux L., Gennis R.B. (1990). Expression of cyoA and cyoB demonstrates that the co-binding heme component of the *Escherichia coli* cytochrome-o complex is in subunit I. J. Biol. Chem..

[26] Nguyen T.Q., Hanh B.T.B., Jeon S., Heo B.E., Park Y., Choudhary A., Moon C., Jang J. (2022). Synergistic effect of Q203 combined with PBTZ169 against. Antimicrob. Agents Chemother..

[27] Pethe K., Bifani P., Jang J., Kang S., Park S., Ahn S., Jiricek J., Jung J., Jeon H.K., Cechetto J. (2013). Discovery of Q203, a potent clinical candidate for the treatment of tuberculosis. Nat. Med..

[28] Kitchen D.B., Decornez H., Furr J.R., Bajorath J. (2004). Docking and scoring in virtual screening for drug discovery: Methods and applications. Nat. Rev. Drug Discov..

[29] Liu P., Li D., Li W., Wang D. (2019). Mitochondrial unfolded protein response to microgravity stress in nematode *Caenorhabditis elegans*. Sci. Rep..

[30] Menke A., Nitschke F., Hellmuth A., Helmel J., Wurst C., Stonawski S., Blickle M., Weiß C., Weber H., Hommers L. (2021). Stress impairs response to antidepressants via HPA axis and immune system activation. Brain Behav. Immun..

[31] Wei P.L., Keller C., Li L.J. (2020). Neuropeptides in gut-brain axis and their influence on host immunity and stress. Comput. Struct. Biotechnol. J..

[32] Pavlov V.A., Tracey K.J. (2017). Neural regulation of immunity: Molecular mechanisms and clinical translation. Nat. Neurosci..

[33] Richmond J.E., Davis W.S., Jorgensen E.M. (1999). UNC-13 is required for synaptic vesicle fusion in. Nat. Neurosci..

[34] Stefanakis N., Carrera I., Hobert O. (2015). Regulatory logic of pan-neuronal gene expression in *C. elegans*. Neuron.

[35] Kirienko N.V., Cezairliyan B.O., Ausubel F.M., Powell J.R. (2014). *Pseudomonas aeruginosa* PA14 pathogenesis in *Caenorhabditis elegans*. Methods Mol. Biol..

[36] Tan M.W., Mahajan-Miklos S., Ausubel F.M. (1999). Killing of *Caenorhabditis elegans* by *Pseudomonas aeruginosa* used to model mammalian bacterial pathogenesis. Proc. Natl. Acad. Sci. USA.

[37] Liu J., Zhang P., Zheng Z., Afridi M.I., Zhang S., Wan Z., Zhang X., Stingelin L., Wang Y., Tu H. (2023). GABAergic signaling between enteric neurons and intestinal smooth muscle promotes innate immunity and gut defense in *Caenorhabditis elegans*. Immunity.

[38] Willing B.P., Russell S.L., Finlay B.B. (2011). Shifting the balance: Antibiotic effects on host-microbiota mutualism. Nat. Rev. Microbiol..

[39] Cash H.L., Whitham C.V., Behrendt C.L., Hooper L.V. (2006). Symbiotic bacteria direct expression of an intestinal bactericidal lectin. Science.

[40] Ivanov I.I., de Llanos Frutos R., Manel N., Yoshinaga K., Rifkin D.B., Sartor R.B., Finlay B.B., Littman D.R. (2008). Specific microbiota direct the differentiation of IL-17-producing T-helper cells in the mucosa of the small intestine. Cell Host Microbe.

[41] Anuforom O., Wallace G.R., Piddock L.V. (2015). The immune response and antibacterial therapy. Med. Microbiol. Immunol..

[42] Bhattarai Y., Muniz Pedrogo D.A., Kashyap P.C. (2017). Irritable bowel syndrome: A gut microbiota-related disorder?. Am. J. Physiol. Gastrointest. Liver Physiol..

[43] Sekirov I., Tam N.M., Jogova M., Robertson M.L., Li Y., Lupp C., Finlay B.B. (2008). Antibiotic-induced perturbations of the intestinal microbiota alter host susceptibility to enteric infection. Infect. Immun..

[44] Etayash H., Alford M., Akhoundsadegh N., Drayton M., Straus S.K., Hancock R.E.W. (2021). Multifunctional antibiotic-host defense peptide conjugate kills bacteria, eradicates biofilms, and modulates the innate immune response. J. Med. Chem..

[45] Von Wulffen J., RecogNice-Team, Sawodny O., Feuer R. (2016). Transition of an anaerobic *Escherichia coli* culture to aerobiosis: Balancing mRNA and protein levels in a demand-directed dynamic flux balance analysis. PLoS ONE.

[46] Zhang J., Li X., Olmedo M., Holdorf A.D., Shang Y., Artal-Sanz M., Yilmaz L.S., Walhout A.J.M. (2019). A delicate balance between bacterial iron and reactive oxygen species supports optimal *C. elegans* development. Cell Host Microbe.

[47] Paganini D., Zimmermann M.B. (2017). The effects of iron fortification and supplementation on the gut microbiome and diarrhea in infants and children: A review. Am. J. Clin. Nutr..

[48] Su C.C., Lyu M., Morgan C.E., Bolla J.R., Robinson C.V., Yu E.W. (2021). A ‘Build and Retrieve’ methodology to simultaneously solve cryo-EM structures of membrane proteins. Nat. Methods.

