# Supplementary material for: Antibiotics Trigger Host Innate Immune Response via Microbiota–Brain Communication in C. elegans
Source: Int J Mol Sci. 2024 Aug 14;25(16):8866. doi: 10.3390/ijms25168866 (PMC11354627; doi:10.3390/ijms25168866)
Supplement: Supplementary file 1 [file ijms-25-08866-s001.zip › ijms-3119986-supplementary.pdf]

Supplementary Materials:

Table S1: List of screening result for *E. coli* K-12 mutants that activate *cyp-14A4* expression of *C. elegans*.

Mutant genes	Relative Fluorescence Intensity
yaiF	282298.0149
fepG	279111.2037
ycgL	260477.3758
yfdL	260139.5916
yeiC	245516.5485
ycfJ	225171.9516
crcB	205460.0761
tfaR	192488.2835
ybjX	186417.0918
ynjF	183578.7997
ybcH	176977.7218
ybeM	174597.4587
ycaP	171789.7532
yfcP	166807.4521
cyoD	159138.6194
ybfQ	156945.7961
ycbB	156571.2284
hyfR	154622.9124
ybfM	149213.7796
ygaM	145989.0675
rstB	142980.5033
hlyE	142368.8805
ygbM	139491.1329
ycfX	134860.2334
cyoA	131388.5909
ygjV	127035.359
yejB	126164.8451
allA	125977.875
cyoB	122005.2006
cyoC	120686.8849
yfiB	116097.6076
mutM	115765.4403
pps	112796.3948
ydeJ	112627.7585

ybaW	107388.2031
yejG	103030.8043
ydiH	90180.80111
ybgE	89446.92455
galS	82926.85279
ypfE	82552.51417
ybeD	81947.31554
ydjA	78496.5056
yciH	77779.87779
ybiB	75930.64369
ycfK	66445.56828
yfdK	65325.03713
yeeD	62175.7023
ybeY	61816.10336
ycbJ	61726.66344
ybhO	56851.78775
hisH	54604.23064
ybhO	52842.21297
btuR	49384.19707
ybfB	45370.82652
abgB	45106.5595
allC	36583.21653
pqiA	33152.72372
mntR	32160.16869
yfcL	31970.0083
yedD	26521.64713
ybhN	24966.65544
yniB	24278.79187
ybaK	24042.60888
ygaD	22562.3956
ydcC	21529.92955
yeeS	19940.93226
eutA	17646.39701
ydfU	15878.9669
rzoR	15199.57178
yliI	11144.88418
yeeN	8709.972321
yaiE	8162.130262
yeiU	7578.739366

ydfD	7513.897348
nac	7374.254306
ybdZ	5348.193842
cheA	314.4257194
BW25113	100

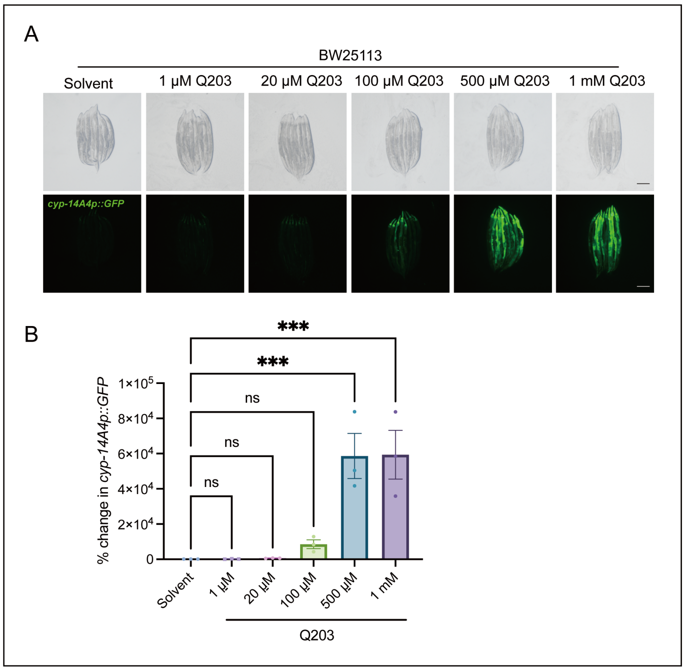

Figure S1. Q203 activates *cyp-14A4* in a concentration-dependent manner. **(A and B)** Images and a bar graph indicating the expression of *cyp-14A4p::GFP* in worms treated with various concentrations of Q203 are presented. The percentage change in *cyp-14A4p::GFP* expression induced by Q203 was determined by normalizing to the levels in worms treated with a solvent control. The data are expressed as the mean ± SEM. Significance was assessed using a one-way ANOVA test (***$p < 0.001$), with a sample size of $n > 20$ for each group. The scale bar in the images corresponds to 200 μm.

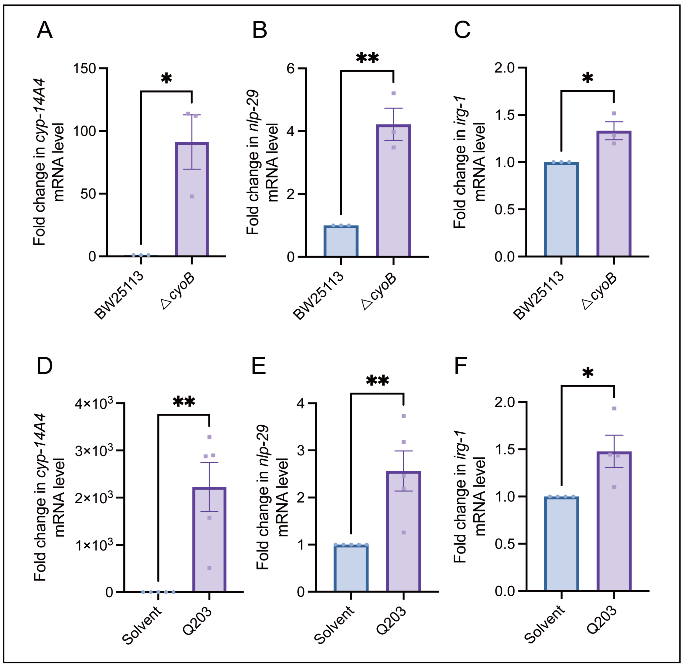

Figure S2. Multiple innate immune-related genes were activated by *ΔcyoB E. coli* and Q203. **(A-F)** qPCR measurements revealed the induction of the transcription of *cyp-14A4*, *nlp-29*, and *irg-1* in *C. elegans*. The fold changes in the transcription of the indicated genes induced by *ΔcyoB E. coli* and Q203 were analyzed by normalizing to the levels in BW25113-treated animals or solvent-control animals. The data are expressed as the mean ± SEM. Significance was assessed using an unpaired t-test (*p < 0.05, **p < 0.01), with a sample size of n > 400 for each group.
